# Study on Mechanism of Invigorating Qi and Promoting Blood Circulation in Treatment of Angiogenesis after Myocardial Infarction Using Network Pharmacology

**DOI:** 10.1155/2022/5093486

**Published:** 2022-05-23

**Authors:** Rui Fan, Yanbo Sui

**Affiliations:** ^1^The School of Graduate, Heilongjiang University of Chinese Medicine, Harbin 150000, China; ^2^Department of Cardiovascular Disease, The First Affiliated Hospital of Heilongjiang University of Traditional Chinese Medicine, Harbin 150000, China

## Abstract

**Objective:**

This article aims to explore the impact and mechanism of invigorating qi and promoting blood circulation (IQPBC) on angiogenesis after myocardial infarction (AMI) by using network pharmacology approach.

**Methods:**

First, IQPBC was searched on the traditional Chinese medicine systems pharmacology database and analysis platform (TCMSP), and the main active ingredients and targets of IQPBC were screened and obtained. Second, by virtue of GeneCards and Online Mendelian Inheritance in Man (OMIM) databases, the targets related to AMI are screened and then obtained. Then, the intersection targets between IQPBC and AMI can be obtained by using online tool Venny 2.1.0. Third, based on the STRING database, the interaction of target proteins is established and some key targets can be analyzed and obtained. Finally, the IQPBC-AMI interaction network is constructed by using Cytoscape, and Gene Ontology (GO) and Kyoto Encyclopedia of Genes and Genomes (KEGG) enrichment analyses are executed by DAVID and OmicShare databases.

**Results:**

143 intersection targets between IQPBC and AMI are obtained. Besides, key active ingredients, namely, quercetin, tanshinone, kaempferol, and luteolin, are shown. Furthermore, AKT1, VEGFA, STAT3, HIF-1*α*, and other 10 key targets are obtained. A total of 752 enrichment results are acquired by using GO analysis. KEGG pathway enrichment analysis shows 241 signaling pathways, focusing on cancer, fluid shear stress and atherosclerosis, and TNF and PI3K/AKT signaling pathways.

**Conclusion:**

This article studies the potential targets and signaling pathways of IQPBC drugs acting on AMI via the network pharmacology approach, which better illustrates the effect and mechanism, and provides some good ideas for the following mechanism research studies.

## 1. Introduction

In recent years, it is reported that cardiovascular disease (CVD), currently, was already the first factor leading to death among urban and rural residents in China. Not just that, the number of CVD patients has reached 290 million [1–4]. As is well known, acute myocardial infarction [5–7] and heart failure [8–10] are the leading causes of CVD and have been received great attention. Numerous medical scientists have made great efforts to research and develop effective medicine for the prevention and treatment of ischemic heart disease [11, 12]. Recent studies have shown that therapeutic angiogenesis can help establish collateral circulation, restore blood oxygen supply to the ischemic myocardium, and improve cardiac function [13, 14]. Fortunately, it was verified that therapeutic angiogenesis has been the potential and effective treatment strategy for ischemic heart disease. And so, it has become a research hotspot in the field of CVD.

Traditional Chinese medicine has shown obvious advantages and potential in promoting angiogenesis. For instance, some effective herbs, components, and compound preparations can promote the growth of coronary artery collateral vessels and protect the ischemic myocardium. Based on this fact, this article considers a Chinese herbal medicine compound: Danshen (*Salvia miltiorrhiza Bunge*), Guizhi (*Ramulus Cinnamomi*), Huangqi (*Radix Astragali*), Mu dan pi (*Cortex Radicis Moutan*), and Renshen (*Radix Ginseng*), which have been verified that all these show good effect in IQPBC and removing stasis [15, 16]. Specifically, due to the advantages of tonifying qi, antiperspirant, water detumescence, and detoxification, Huangqi is widely used in clinical practice, which can not only warm the spleen and lungs but also relieve exterior syndrome by diaphoresis. Guizhi has the ability to promote sweating, prevent cold, and treat palpitation and arrhythmia. Danshen and Mudan pi both have the effect of promoting blood circulation and removing blood stasis and are often used for chest paralysis and heart pain. Moreover, Mudan pi can help sleep and quickly achieve the effect of lowering blood pressure. Renshen is known to tonify the qi and blood, nourish the heart, calm the mind, and refresh the brain. It can improve immune function, strengthen the heart, resist shock, and so on. Clinically, Renshen and Huangqi are jointly utilized to improve the therapeutic effect. Up to now, lots of remarkable research studies have confirmed that the IQPBC compound can significantly increase the microvascular density of the ischemic myocardium, enhance VEGF transcriptional activity and phosphorylation levels of JAK and STAT3 [17], promote angiogenesis, and improve cardiac function. Although some remarkable results with regard to IQPBC acting on AMI have been reported [18–20], the action mechanism of IQPBC in the treatment of AMI is still not clearly illustrated.

Network pharmacology [21] is a fresh discipline that emphasizes the multipathway regulation of signaling pathways, improves the therapeutic effect of drugs, and reduces toxic and side effects, thus improving the success rate of clinical trials of new drugs and saving the research cost of drugs [22]. Thus, inspired by the advantages of network pharmacology, this article aims to further explore key targets and signaling pathways of the IQPBC compound. Then, this article can provide a reference for the following study on mechanism of action and pharmacodynamic substances.

**Figure 1:**
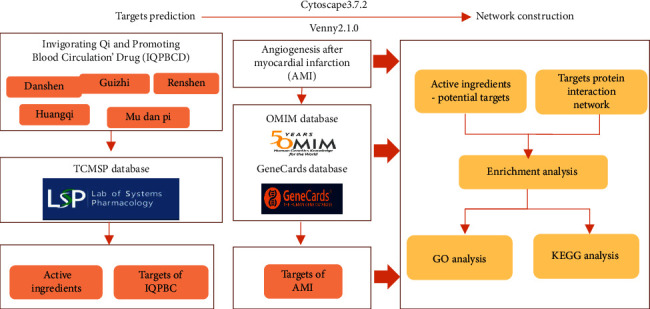
Framework of the systematic strategies to elucidate the mechanisms of IQPBC in the treatment of AMI.

The main work of this article is summarized as follows: First, the considered Chinese herbal compound is searched on the TCMSP [23] database to screen the active ingredients and corresponding targets. Second, we search and screen the targets of AMI using OMIM [24] and GeneCards databases [25]. Third, we obtain the intersection targets of IQPBC and AMI by using online tool Venny 2.1.0. The protein-protein interaction (PPI) network is obtained from the STRING database [26] and visualized in Cytoscape 3.7.2 software, and the key targets are screened by calculating the degree of all intersection targets. Finally, the “IQPBC-AMI- active ingredient-target” network is constructed and visualized in Cytoscape 3.7.2 [27], and then, GO and KEGG enrichment analyses [28, 29] are performed. The framework of the systematic strategies to elucidate the mechanisms of IQPBC in the treatment of AMI is shown in Figure 1.

## 2. Materials and Methods

### 2.1. Active Ingredients and Targets of IQPBC

The objective of TCMSP (https://old.tcmsp-e.com/tcmsp.php) is to capture the relationships between drugs, targets, and diseases. Based on TCMSP, the main active ingredients and corresponding targets of drugs can be screened by limiting oral bioavailability (OB) ≥30% and drug-likeness (DL) ≥0.18. Meanwhile, the UniProt database was used for data correction.

### 2.2. Targets of AMI

Targets for AMI are screened using the OMIM database (https://omim.org/) and GeneCards database. First, enter the OMIM database, click “Gene Map,” search with “angiogenesis of myocardial infarction” as the search term, and download the search results as Excel files. Second, the GeneCards database was used to search for “angiogenesis of myocardial infarction,” and the search results were downloaded as Excel files. Finally, combined with the aforementioned results, the corresponding tables of IQPBC and AMI targets were imported by using the online tool Venny 2.1.0 (https://bioinfogp.cnb.csic.es/tools/venny/index.html), and then the intersection targets of IQPBC and AMI were obtained.

### 2.3. Construction and Analysis of “IQPBC-AMI-Active Ingredient-Target” Network

The obtained intersection targets and active ingredients were imported into Cytoscape 3.7.2 software to analyse and explore the intrinsic correlations. The information in the “Target Table Data” option indicates that the imported node attribute tables will be associated with the previous network diagram, where “Network Collection” is the network file previously imported. The other parameters are defaulted as follows and unchanged, if there are other options, then go to the dropdown list and select them again. In the “Preview” option, gene, name, molecular type, degree, and the first gene column are set to “Key” to ensure that the gene id is not repeated, the second, third, and fourth columns represent attributes, and click “OK.”

### 2.4. Construction of the PPI Network

First, import the intersection targets into the STRING database to construct the PPI network with “Homo Sapiens” as filter conditions. Meanwhile, set the required minimum interaction score as 0.4, and hide the unconnected nodes. Finally, download the TSV files and import them into Cytoscape 3.72 for visualization. In this study, it is worth mentioning that the Markov cluster (MCL) algorithm in the STRING database and the CytoHubba plugin in Cytoscape 3.7.2 software were jointly utilized to obtain the clusters and key targets.

### 2.5. GO and KEGG Enrichment Analyses

DAVID (https://david.ncifcrf.gov/) can be used for differential analysis of genes and enrichment of pathways, and it links genes in the input list to biological annotations. The steps can be summarized as follows: enter the “Function Annotation Table” and upload the intersection targets. Then, click “OFFICIAL_GENE_SYMBOL,” “Gene List,” “Submit List,” and “Homo sapiens” in turns. Next, select “Gene-Ontology” and “Kegg-Pathway” and download the files, respectively. In the end, combined with R software, DAVID, and OmicShare, the GO and KEGG enrichment analyses of intersection targets can be obtained.

## 3. Results

### 3.1. Active Ingredients and Targets of IQPBC

**Figure 2:**
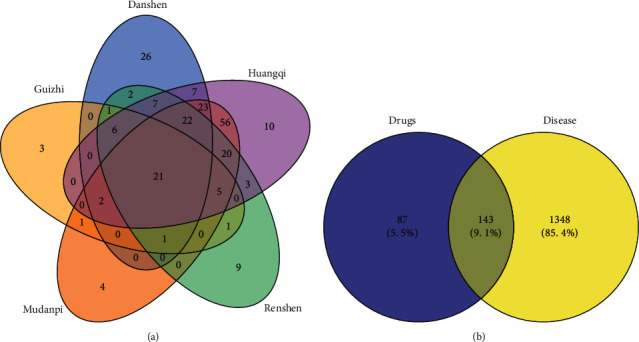
Venny diagrams of (a) the intersection targets of all drugs in IQPBC and (b) the intersection targets between IQPBC and AMI.

By utilizing the TCMSP database and adopting constraint conditions OB ≥30% and DL ≥0.18, 125 active ingredients are screened, which contain 65 in Danshen, 20 in Huangqi, 11 in Mu dan pi, 22 in Renshen, and 7 in Guizhi, and after removing the active ingredients lacking target prediction information, 96 active ingredients and 1953 targets are finally obtained. 1672 targets can be successfully converted in the UniProt database, and 230 drug targets are obtained after weight removal. The Venny diagram of intersection targets of IQPBC is shown in Figure 2(a).

### 3.2. Intersection Targets between IQPBC and AMI

**Figure 3:**
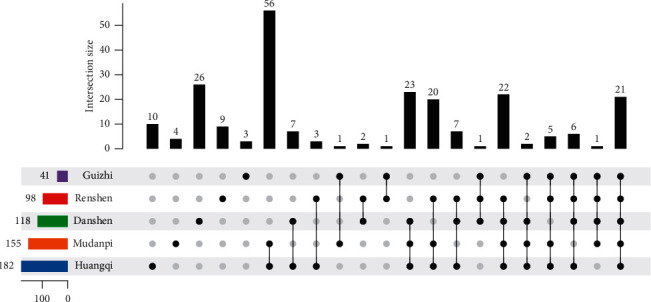
Venny diagram of intersection targets between IQPBC and AMI.

By deprocessing the results retrieved and screened in the OMIM database and GeneCards database, 1491 disease targets can be obtained. Then, importing the 230 drug targets and 1491 disease targets into the online tool Venny 2.1.0, we obtain 143 intersection targets, and the Venny diagram of intersection targets is shown in Figure 2(b). In order to clearly demonstrate the intersection targets of IQPBC, Figure 3 is drawn; it can be seen from the bottom left of the figure that the targets of Guizhi, Renshen, Danshen, Mu dan pi, and Huangqi are 41, 98, 118, 155, and 182, respectively.

### 3.3. “IQPBC-AMI-Active Ingredient-Target” Network

**Figure 4:**
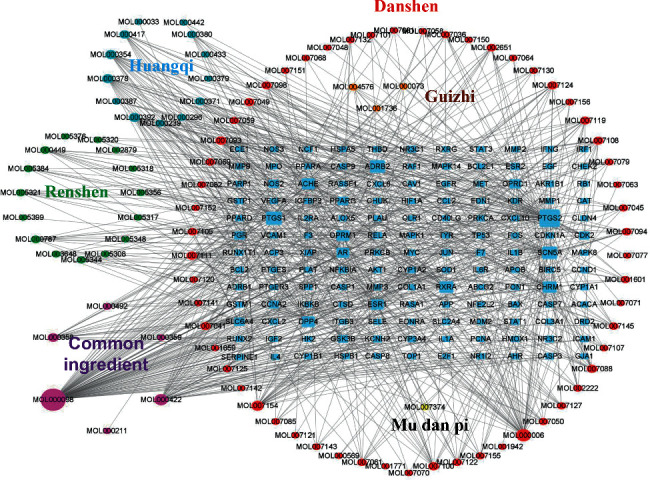
IQPBC-AMI-active ingredients-targets network.

According to the obtained active ingredients, targets of IQPBC, and targets of AMI, we construct the “IQPBC-AMI-active ingredient-target” network. As shown in Figure 4, 238 nodes and 821 edges are presented, the square nodes represent potential genes, and the round nodes represent active ingredients. The area of a node represents its degree. The larger the area is, the more important the node is in the network. If the two nodes are connected by an edge, it indicates that there is an interaction between the target proteins. By analysing the constructed network, the significance of active ingredients can be ordered as quercetin, luteolin, kaempferol, tanshinone IIA, 7-O-methylisomucronulatol, formononetin, isorhamnetin, beta-sitosterol, dan-shexinkum d, and isotanshinone II. In Table 1, the degree, average shortest path length (ASPL), closeness centrality (CC), and betweenness centrality (BC) are shown.

### 3.4. PPI Network Analysis

**Figure 5:**
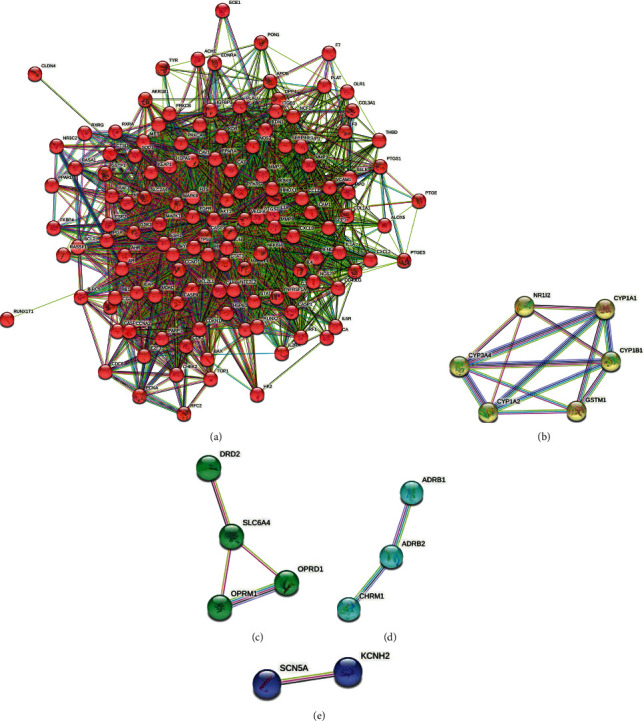
5 clusters in the PPI network by using MCL. The 5 clusters contain 127, 6, 4, 3, 2 gene, respectively.

**Figure 6:**
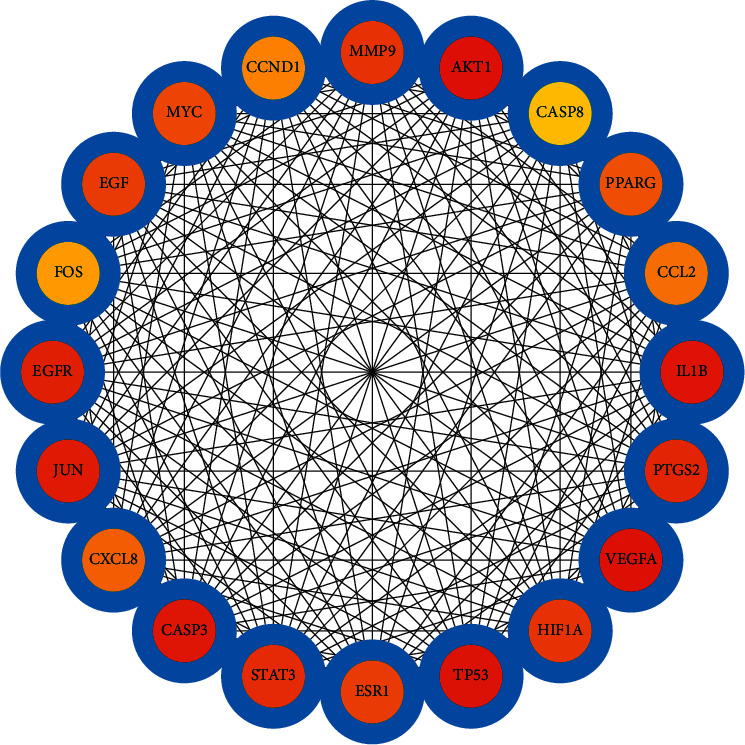
Key targets in the PPI network (the first 20).

**Figure 7:**
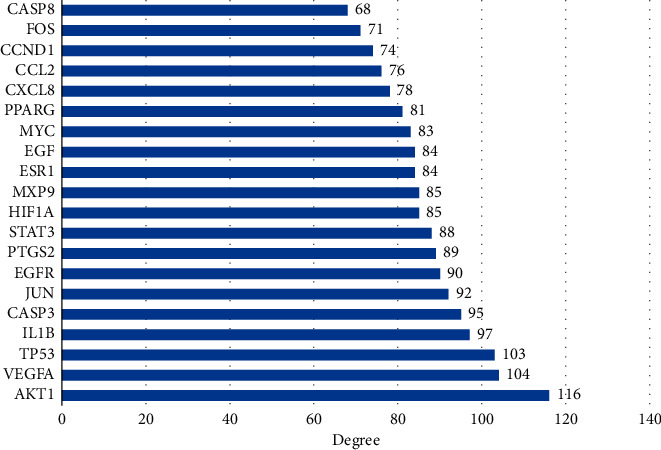
Bar plot of key targets in the PPI network (the first 20).

Based on the obtained intersection targets of IQPBC and AMI, the PPI network is constructed by using the STRING database. By analyzing the whole PPI network, 143 nodes and 2802 edges are obtained. The average degree of its node value is 39.2, and the average local clustering coefficient is 0.644. Based on the Markov MCL algorithm in the STRING database, the constructed PPI network can be divided into 5 clusters, which are shown in Figure 5. Then, we calculate the degree by using the CytoHubba plugin in Cytoscape 3.7.2 software and obtain the key targets (degree >90) as follows: AKT1, VEGFA, TP53, IL1B, CASP3, JUN, EGFR, PTGS2, STAT3, and HIF-1*α*. The first 20 key targets are shown in Figure 6. In order to better show the key targets to the readers, a bar plot of the first 20 key targets is shown in Figure 7.

### 3.5. GO Enrichment Analysis

**Figure 8:**
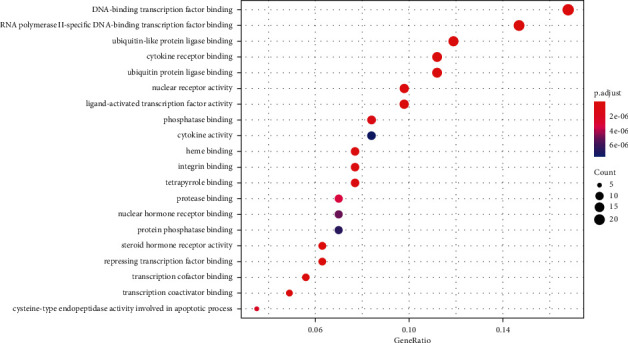
Dot plot of GO enrichment analysis. Note: gene ratio (*X*-axis) and GO term (*Y*-axis). The different sizes of the bubbles represent the count of genes and the diffuse color of the bubbles represents the importance of genes.

**Figure 9:**
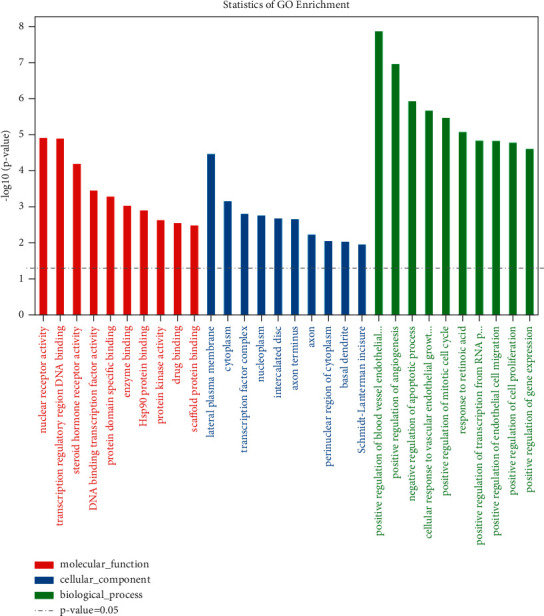
Bar plot of BP, CC, and MF in GO enrichment analysis. Note: GO terms (*X*-axis) and log10 (*p* value) (*Y*-axis). The higher the bar graph height, the smaller the corresponding *p* value.

**Figure 10:**
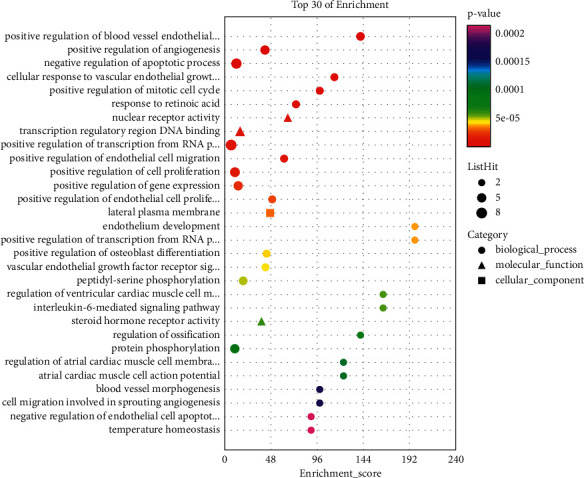
Dot plot of GO enrichment function analysis includes MF, CC, and BP. Note: enrichment score (*X*-axis) and GO terms (*Y*-axis). Different shapes represent different GO classifications (BP, CC, and MF). The size of the point denotes the number of different genes in GO terms. The bubble color varies from purple-blue-green-red, and the smaller the enrichment *p* value, the greater the significance.

GO enrichment analysis shows that 752 terms are obtained, which includes 575 biological process (BP) terms, 110 molecular function (MF) terms, and 67 cellular components (CC) terms. In Figure 8, the outcomes of GO enrichment analysis (the first 20) are shown, which include DNA-binding transcription factor binding, RNA polymerase II-specific DNA-binding transcription factor binding, ubiquitin-like protein ligase binding, cytokine receptor binding, ubiquitin protein ligase binding, nuclear receptor activity, ligand-activated transcription factor activity, phosphatase binding, tetrapyrrole binding, integrin binding, heme binding, repressing transcription factor binding, steroid hormone receptor activity, transcription cofactor binding, and transcription coactivator binding. In order to better show the results of GO enrichment analysis, a dot plot diagram and a bar plot are drawn for the items of biological process, cell composition, and molecular function in GO enrichment analysis results, which are shown in Figures 9 and 10, respectively. From Table 2, it can be seen that the detailed GO enrichment analysis outcomes (the first 10).

### 3.6. KEGG Pathway Analysis

**Figure 11:**
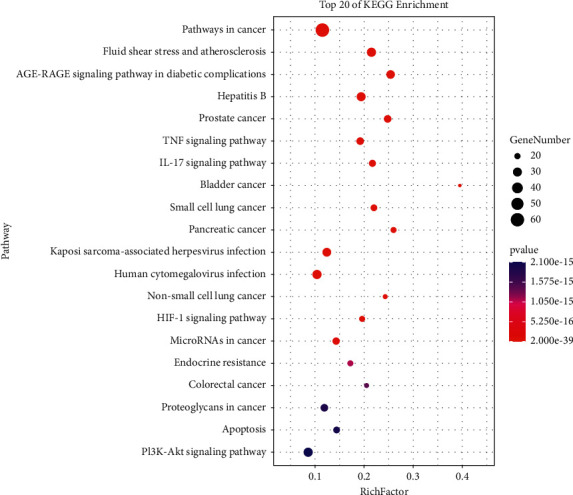
Dot plot of KEGG pathway analysis. Note: pathway (*Y*-axis), rich factor (*X*-axis), and *p* value (color). The larger the point is, the more genes fall into this pathway. The bubble color changes from purple-blue-green-red, and the *p* value of enrichment is smaller, which indicates a significant degree.

**Figure 12:**
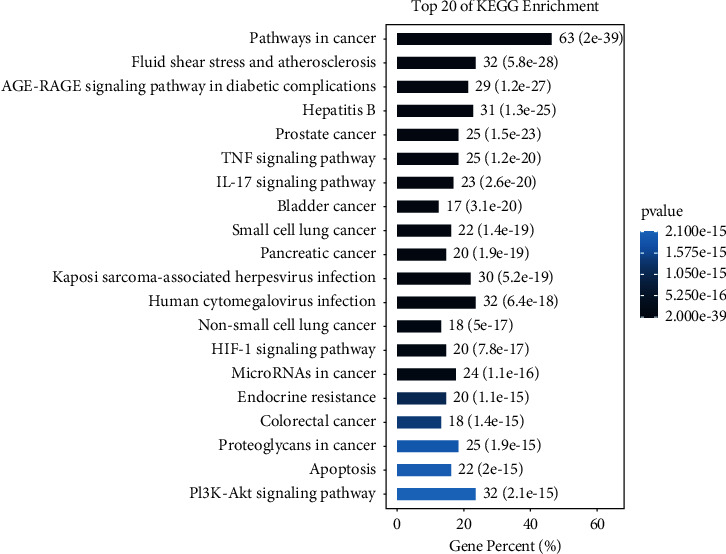
Bar plot of KEGG pathway analysis. Note: gene percent (*X*-axis) and pathway (*Y*-axis).

**Figure 13:**
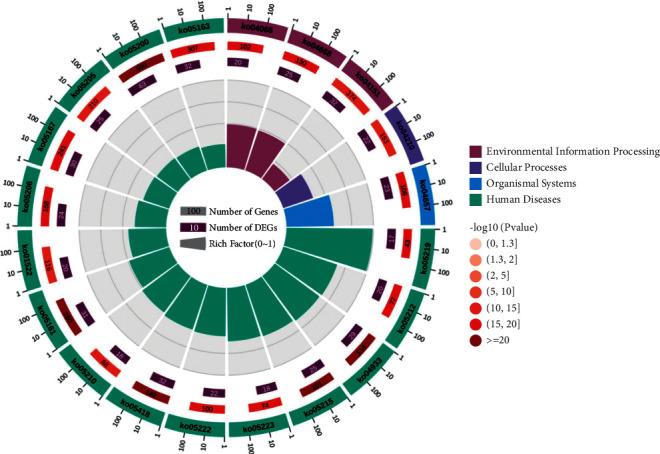
Enrichment of graph.

KEGG pathway analysis shows 241 treatment pathways are screened (*p* value <0.05). The first 20 pathways are shown in Figures 11 and 12. From Table 3, it can be seen that the detailed pathways contain the following: pathways in cancer, fluid shear stress and atherosclerosis, AGE-RAGE signaling pathway in diabetic complications, hepatitis B, prostate cancer, TNF signal pathway, IL-17 signaling pathway, bladder cancer, small-cell lung cancer, pancreatic cancer, Kaposi sarcoma-associated herpesvirus infection, human cytomegalovirus infection, non-small-cell lung cancer, HIF-1*α* signaling pathway, microRNAs in cancer, endocrine resistance, colorectal cancer, proteoglycans in cancer, apoptosis, and PI3K-Akt signaling pathway. The enriched classification, the number and *p* values of the classification, the prospective gene, and Rich Factor values are shown in Figure 13.

## 4. Discussion

As we know, angiogenesis has the ability to restore blood oxygen supply and protect cardiac function and has been widely investigated. Furthermore, network pharmacology of traditional Chinese medicine (TCM) takes integrity and systematicness as the starting point to construct a complex network of component-target-disease to analyze and clarify the mechanism of action of the research object [30, 31], which is matched with the holistic concept of TCM and the principle of treatment that is based upon syndrome differentiation [32]. Inspired by the advantage of the network pharmacology approach, this article aims to analyze the relationship among IQPBC, targets, and AMI [33] via the network pharmacology approach and predict the active ingredients and potential intersection targets.

By analyzing in detail, four key active ingredients of IQPBC in the treatment of AMI are screened and obtained, namely, quercetin, tanshinone, kaempferol, and luteolin. As a potential inhibitor of angiogenesis, quercetin plays a vital part in anti-inflammatory and antiatherosclerosis. Furthermore, some experiments have shown that quercetin can significantly recover the blood flow of the ischemic hind limb in mice, increase the capillary density of the ischemic muscle, and improve cardiac function [34]. Tanshinone can protect the heart and stabilize atherosclerotic plaque by reducing the inflammatory response after myocardial infarction. Under hypoxia, luteolin can promote angiogenesis by mediating HIF-1*α* and STAT3 signaling pathways [35]. Kaempferol has a protective effect on cardiovascular function [36, 37], which inhibits HIF-1*α* and VEGF2 activation in endothelial cells mainly through ERK/p38 MAPK and PI3K/Akt/mTOR [38, 39] and then regulates angiogenesis.

Based on the obtained results, it can be seen from the PPI network that AKT1, VEGFA, STAT3, and HIF-1*α* are the key targets of IQPBC in the treatment of AMI. AKT1 shows a great effect on growth factor signal transduction, cell proliferation, differentiation, and transcriptional regulation and development. It is worth noting that STAT3 is considered a key regulator for adjusting the cardiac microenvironment [40] where it can regulate the secretion of cardiac muscle cells, endothelial cells, and heart cells and affect the communication between myocardial cells. Recent studies have shown that IQPBC can adjust coronary ligation in rats at the myocardial STAT3 phosphorylation level, increase the ischemic myocardial vascular density, and improve heart function [17]. VEGFA promotes angiogenesis through activation of VEGFR2 and its downstream signal cascade [41]. Under hypoxia, the infarcted myocardium releases a large amount of HIF-1*α*, which activates HIF-1 to bind to VEGF and activate the VEGFA/VEGFR2 signaling pathway. Angiogenesis is induced by PKC, NOS, Akt, and other pathways. Combined with the relevant literature, GO and KEGG enrichment analyses are directed to find that the PI3K-AKT signaling pathway, TNF signaling pathway, and fluid shear stress and atherosclerosis signaling pathway are the key pathways of IQPBC in the treatment of AMI. The PI3K-Akt signaling pathway has the ability to improve cardiac function and myocardial ischemia after MI by ensuring the survival, proliferation, and differentiation of endothelial cells. Some remarkable results have found that AngII and its receptor PI3K/AKT signaling pathway may be related to AMI [42, 43], and some comparative experiments have confirmed that AngII may activate the PI3K/AKT signaling pathway by promoting AKT phosphorylation and angiogenesis [44, 45].

Tumor necrosis factor (TNF) is expressed in ischemic tissues and promotes angiogenesis [46, 47]. Under hypoxia, VEGF expression is upregulated, which breaks the balance between proangiogenic and inhibitory factors, thus promoting angiogenesis in the infarcted myocardium and preventing ventricular remodeling. It has been found that the inflammatory factor TNF-*α* is highly expressed in the myocardial tissue after myocardial infarction, and TNF-*α* stimulates the expression of the stem cell factor (SCF) in myocardial cells, thereby inducing the migration of BMSC from the damaged human myocardium and differentiation into cardiac cells, enhancing the repair of the damaged heart and improving the function of the heart [48]. Fluid shear stress, the most important factor in atherosclerosis, increases the activity of many kinases that regulate the phosphorylation of many signal transduction proteins in endothelial cells, thereby regulating vascular structure and function. Studies have found that when endothelial cells are subjected to shear stress, they inhibit IL-6-induced STAT3 phosphorylation in a shear stress-dependent manner and reduce STAT3 nuclear translocation and STAT3 binding to DNA [49]. Laminar shear stress inhibits endothelial cell proliferation by inhibiting STAT3 activation.

## 5. Conclusions

In conclusion, quercetin, tanshinone, kaempferol, and luteolin in IQPBC act on AKT1, VEGFA, STAT3, HIF-1*α*, and other targets as key components. It may regulate PI3K/AKT signaling pathway, TNF signaling pathway, and fluid shear stress and atherosclerosis signaling pathway to promote angiogenesis after infarction. It laid the foundation for the mechanism and pharmacodynamic substance of IQPBC in the treatment of AMI. Therapeutic angiogenesis plays an important role in restoring revascularization and improving cardiac function, which opens a new perspective for the treatment of myocardial infarction.

## Figures and Tables

**Table 1:** Statistics of “IQPBC-active ingredients-targets.”

ID	Name	ASPL	CC	Degree	BC
MOL00098	Quercetin	1.8101	0.5524	105	0.4924
MOL00006	Luteolin	2.3839	0.4184	41	0.1032
MOL00042	Kaempferol	2.3670	0.4224	34	0.0748
MOL007154	Tanshinone IIA	2.4936	0.4010	27	0.0525
MOL000378	7-0-Methylisomucronulatol	2.5189	0.3969	20	0.0143
MOL000392	Formononetin	2.5247	0.3956	19	0.0142
MOL000354	Isorhamnetin	2.5274	0.3956	19	0.0246
MOL000358	Beta-sitosterol	2.5358	0.3943	17	0.0216
MOL007093	Dan-shexinkun d	2.5443	0.3930	17	0.0080
MOL007111	Isotanshinone II	2.5527	0.3917	16	0.0064

**Table 2:** Results of GO enrichment analysis.

ID	Description	Count	Target name
GO:0004879	Nuclear receptor activity	14	RXRA/PGR/NR3C2/ESR1/AR/PPARG/ESR2/NR3C1/STAT3/NR1I2/PPARD/AHR/PPARA/RXRG
GO:0098531	Ligand-activated transcription factor activity	14	RXRA/PGR/NR3C2/ESR1/AR/PPARG/ESR2/NR3C1/STAT3/NR1I2/PPARD/AHR/PPARA/RXRG
GO:0140297	DNA-binding transcription factor binding	24	RXRA/ESR1/PPARG/RELA/RB1/JUN/NFKBIA/PCNA/MAPK14/GSK3B/STAT3/BCL2/FOS/MYC/NR1I2/PPARD/STAT1/HIF1A/PRKCB/HSPB1/NFE2L2/PARP1/PPARA/RUNX2
GO:0061629	RNA polymerase II-specific DNA-binding transcription factor binding	21	RXRA/ESR1/PPARG/RELA/RB1/JUN/NFKBIA/PCNA/MAPK14/GSK3B/STAT3/FOS/NR1I2/PPARD/STAT1/HIF1A/PRKCB/HSPB1/NFE2L2/PARP1/PPARA
GO:0003707	Steroid hormone receptor activity	9	RXRA/PGR/NR3C2/ESR1/ESR2/NR3C1/PPARD/PPARA/RXRG
GO:0005126	Cytokine receptor binding	16	VEGFA/IL6R/CASP3/IFNG/IL4/CD40LG/STAT3/ITGB3/CASP8/STAT1/IL1B/CCL2/CXCL8/IL1A/CXCL2/CXCL10
GO:0044389	Ubiquitin-like protein ligase binding	17	SCN5A/RELA/EGFR/CDKN1A/RB1/JUN/TP53/NFKBIA/MDM2/GSK3B/KCNH2/BCL2/CASP8/STAT1/HIF1A/HSPA5/CHEK2
GO:0031625	Ubiquitin protein ligase binding	16	SCN5A/RELA/EGFR/CDKN1A/RB1/JUN/TP53/NFKBIA/MDM2/GSK3B/KCNH2/BCL2/CASP8/HIF1A/HSPA5/CHEK2
GO:0001223	Transcription coactivator binding	7	PGR/ESR1/AR/RELA/PPARD/AHR/PPARA
GO:0001221	Transcription cofactor binding	8	PGR/ESR1/AR/RELA/PPARD/AHR/NFE2L2/PPARA

**Table 3:** Results of KEGG enrichment analysis.

ID	Description	Count	Degree	Gene annotation
ko05200	Pathways in cancer	63	20	550 (6.46%)
ko05418	Fluid shear stress and atherosclerosis	32	8	149 (1.75%)
ko04933	AGE-RAGE signalling pathway in diabetic complications	29	7	114 (1.34%)
ko05161	Hepatitis B	31	12	160 (1.88%)
ko05215	Prostate cancer	25	10	101 (1.19%)
ko04668	TNF signalling pathway	25	12	130 (1.53%)
ko04657	IL-17 signalling pathway	23	5	106 (1.25%)
ko05219	Bladder cancer	17	8	43 (0.51%)
ko05222	Small-cell lung cancer	22	8	100 (1.17%)
ko05212	Pancreatic cancer	20	10	77 (0.9%)
ko05167	Kaposi sarcoma-associated herpesvirus infection	30	15	241 (2.83%)
ko05163	Human cytomegalovirus infection	32	13	307 (3.61%)
ko05223	Non-small-cell lung cancer	18	10	74 (0.87%)
ko04066	HIF-1 signalling pathway	20	12	102 (1.2%)
ko05206	MicroRNAs in cancer	24	6	168 (1.97%)
ko01522	Endocrine resistance	20	7	116 (1.36%)
ko05210	Colorectal cancer	18	12	88 (1.03%)
ko05205	Proteoglycans in cancer	25	13	210 (2.47%)
ko04210	Apoptosis	22	68	153 (1.8%)
ko04151	PI3K-Akt signalling pathway	32	67	374 (4.39%)

## Data Availability

All datasets used to support the findings of this study are included within this study.
